# Primary small intestine mesenteric low-grade fibromyxoid sarcoma with foci of atypical epithelioid whorls and diffuse DOG1 expression: a case report

**DOI:** 10.1186/s13000-019-0905-2

**Published:** 2020-03-13

**Authors:** Jialing Huang, Steven Cohen, Gerorge Jour

**Affiliations:** 1 Department of Pathology, Langone Medical Center, New York University, 550 1st Avenue, New York, NY 10016 USA; 2 Department of Surgery, Langone Medical Center, New York University, 550 1st Avenue, New York, NY 10016 USA

**Keywords:** Low grade fibromyxoid sarcoma, Epithelioid, Small bowel, Mesentery, DOG-1

## Abstract

**Background:**

Low-grade fibromyxoid sarcoma (LGFMS) is a rare fibroblastic tumor often involving deep tissue of trunk and lower extremities in young to middle-aged patients. Rarely, LGFMS can occur in other sites including head and neck, chest, abdomen and female reproductive system. Three cases of LGFMS in mesentery of small intestine have been reported and all have conventional histologic features. Herein we reported a unique case of LGFMS in mesentery of small intestine.

**Case presentation:**

A 43 year-old male with chief complaint of lower back pain for 4 years presented to our hospital. Physical exam reveal a firm, non-tender, non-distended, mobile large abdominal mass, which was shown on abdominal CT as a 10 cm retroperitoneal tumor. Biopsy revealed a spindle cell neoplasm in a myxoid background with a delicate vascular network. Tumor resection was performed. Gross examination of the resected specimen showed a 10.8 cm, tan-white, smooth, firm, lobulated mesenteric mass with bulging and gelatinous cut surface and confined within small bowel serosa. Microscopic examination demonstrated foci epithelioid cords and whorls with prominent atypia, in additional of regular, bland-appearing spindle cells in a fibrous and myxoid stroma and osseous metaplasia. The tumor cells stained diffusely positive for DOG1 with moderate staining density, and diffusely and strongly positive for MUC4. Rearrangement involving FUS (16p11.2) gene was identified with break-apart probe and confirmed by Anchored Multiplex PCR. A final diagnosis of low-grade fibromyxoid sarcoma was rendered.

**Conclusion:**

Our case highlights the importance of including LGFMS in the differential diagnosis of mesenteric tumors and the DOG1 positivity which could represent a potential diagnostic pitfall.

## Introduction

Low-grade fibromyxoid sarcoma (LGFMS), also as known as Evans’ tumor, is a rare fibroblastic tumor. It was first described by Evans in 1987 [1]. The tumor most often involves deep tissue of trunk and lower extremities, especially thigh, in young to middle-aged adults with male predominance in all age groups [2]. Less frequently, LGFMS can occur in abdomen (mesentery, intestine) [3–7] and other sites such as female reproductive system (breast, vagina, vulva, broad ligament) [8–12].

LGFMS is an aggressive low-grade tumor typically composed of deceivingly bland spindle-shaped fibroblast cells residing in variably fibrous/myxoid stroma. The tumor usually grow slowly with infiltration with a propensity for late metastatic potential. The tumor cells have palely eosinophilic cytoplasm and round to ovoid nuclei. Pleomorphic Nuclei, nucleoli and mitotic figures are usually absent [13].

LGFMS can display some variable, focal morphologic features in addition to conventional alternating areas of giant rosettes and hypercellularity. These features include epithelioid morphology, hyalinization, cyst degeneration, calcification/osseous metaplasia, multinucleated giant cells, nuclear palisading, nuclear pleomorphism, and tumor necrosis [14–17]. These histologic variations can be misleading. Especially, the giant rosettes featuring central accumulation of collagen and peripheral palisading epithelioid fibroblastic cells can mimic sclerosing epithelioid fibrosarcoma (SEF). Morphologically, SEF differs from LGFMS in two aspects. First, the tumor cells of SEF are epithelioid cells with clear or eosinophilic cytoplasm, forming nests and cords. Second, the stroma is densely sclerotic. SEF is more aggressive than LGMFS in that local recurrences and distant metastases are seen in more than half of cases [18].

MUC4 is a nearly 100% sensitive and specific marker for LGFMS [19]. Notably, MUC4 expression can also be seen in SEF, synovial sarcomas, ossifying fibromyxoid tumors, epithelioid gastrointestinal stromal tumors and myoepithelial carcinomas [14, 19]. LGFMS can also demonstrate positivity of CD99 and BCL-2 [4]. Interestingly, about 40% of LGFMS cases display patchy expression of DOG1 (discovered on GIST-1) with variable intensity; however, no extensive strong positivity has been ever observed.

Cytogenetically, more than 90% of LGFMS harbor t (7;16)(q34;p11), resulting in FUS-CREB3L2 fusion [20–22]. Alternatively, t (11;16)(p11;p11) translocation resulting in EWSR1-CREB3L1 is seen in less than 10% of cases [23, 24]. Of note, MUC4-negative LGFMS with FUS-CREB3L2 fusion has been described [25].

Pathology of small intestine mesenteric LGMFS have been described in 3 cases from 3 different case reports in English literature. The results reveal conventional histopathologic features of all these 3 cases [3, 26, 27]. All showed conventional morphology, no DOG1 expression, nor epithelioid cells.

Herein we reported a case of LGMFS arising in small intestine mesentery with unconventional epithelioid cords and whorls and extensive, diffuse positivity for DOG-1. Our case highlight the diagnostic challenges of such occurrence.

## Case report

**Fig. 1:**
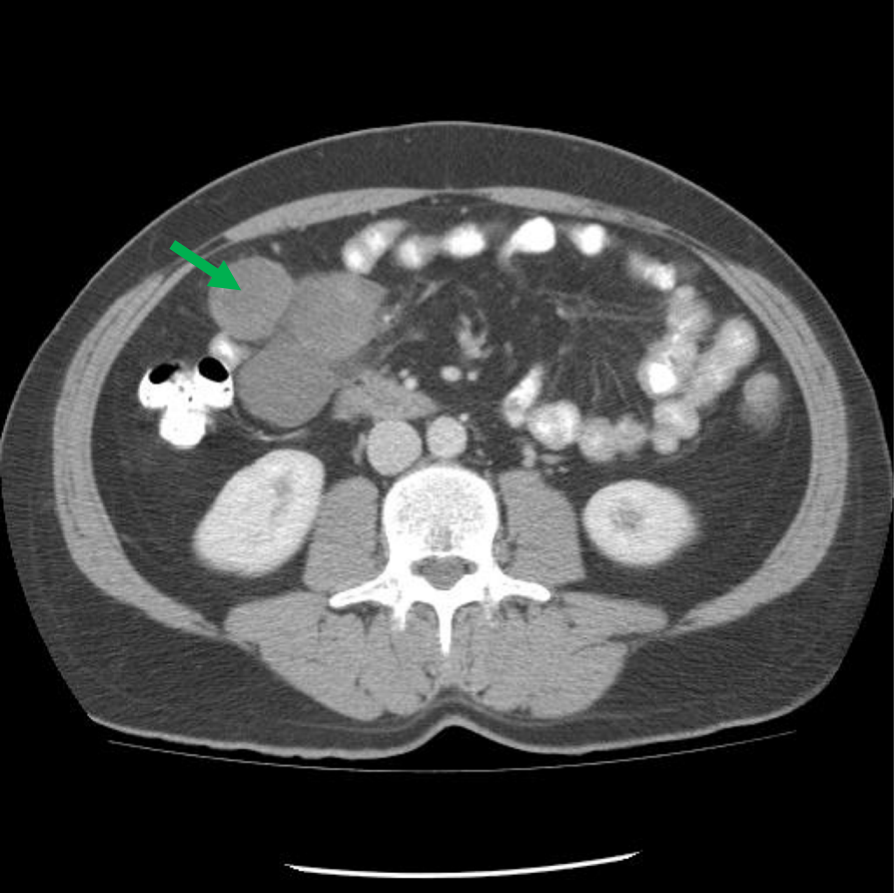
Abdominal CT showed a 10 cm retroperitoneal mesentery mass (put arrows on the mass)

A 43 year-old male who presented with lower back pain for 4 years, loss of 40 pounds in 17 months and urinary urgency for one month. His past medical history was significant for Bell’s palsy, hyperlipidemia, osteoarthritis of cervical spine and vertigo. Physical exam results were normal except a firm, non-tender, mobile large mass palpated in right side of abdomen. The mass was nondistended. Abdominal CT showed a 10 cm retroperitoneal mass (Fig. 1). Laboratory results showed normal hematologic, biochemical and coagulatory results.

### Pathologic findings

Biopsy revealed a spindle cell neoplasm in a myxoid background with a delicate vascular network. The tumor cells showed small oval nuclei with some cytological atypia including hyperchromatism and angulate nuclei. No mitotic figures nor necrosis was noted. Immunohistochemical studies demonstrated that negativity of SMA, desmin, β-catenin, S100, SOX10, AE1/3, EMA, CD117 and CD34 in the tumor cells. DOG1 stain showed rare nonspecific staining in the tumor cells. Proliferative index was low (< 5%) on ki-67 stain (Data not shown). Further molecular classification was attempted but hindered due to the limited specimen.

**Fig. 2:**
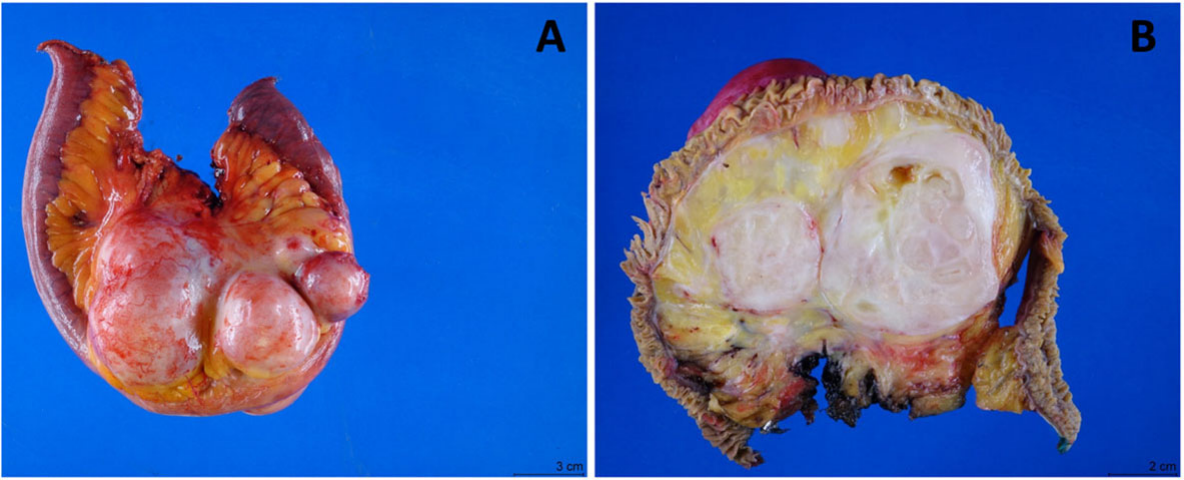
Small intestine mesenteric mass had lobulated outer surface (**a**) and bulging and gelatinous cut surface with focal cystic degeneration (**b**)

Tumor resection was performed with the entire tumor removed. At intraoperative examination, retroperitoneal mass was found clearly intimate with the mesentery and adjacent small bowel showed that the. Gross examination of the resected specimen showed an intact 10.8 × 9.0 × 8.3 cm, tan-white, smooth, firm, lobulated mesenteric mass, which was completely confined within small bowel serosa and had bulging and gelatinous cut surface. The mass was predominately solid (90%) and focally cystic (10%) with a 2.0 × 1.2 × 1.0 cm calcified area (Fig. 2).

**Fig. 3:**
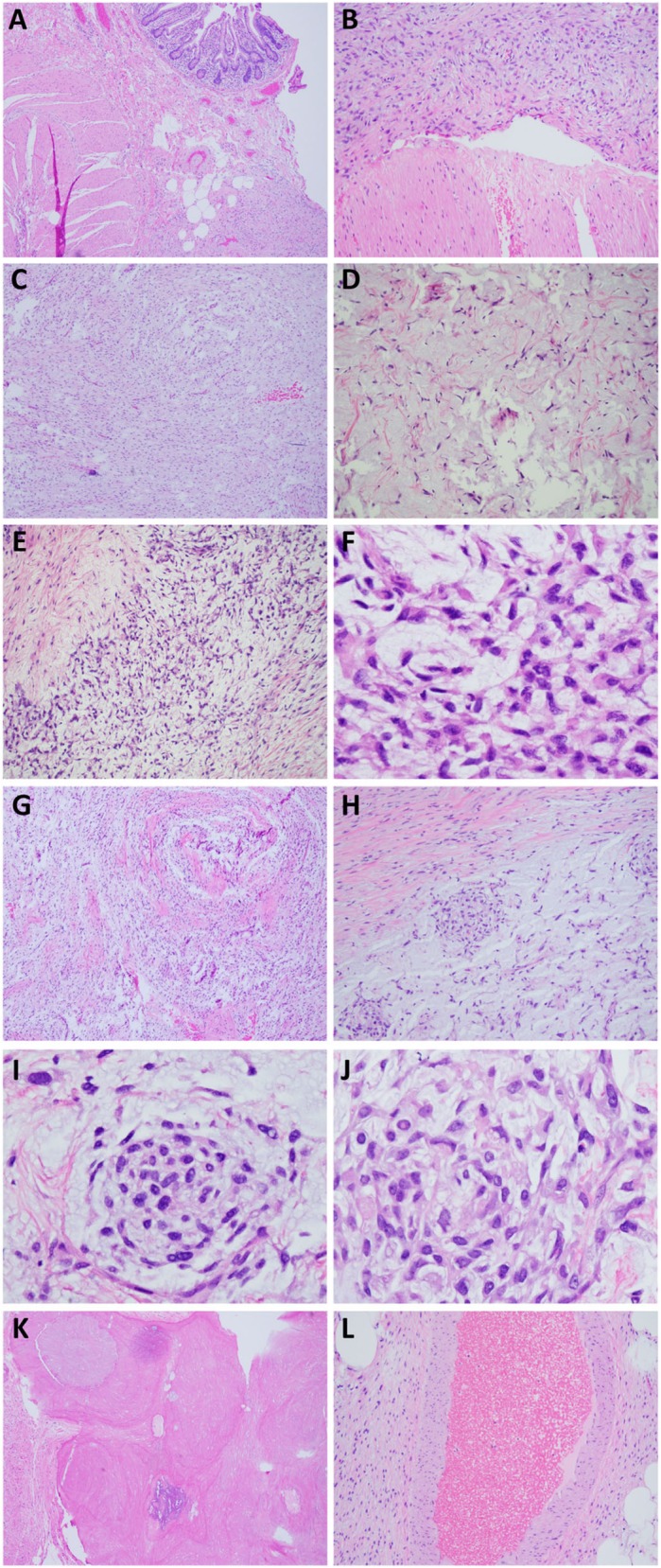
Invasive tumor composed of spindle and epitheloid tumor cells. The tumor invaded muscularis propia (**a**, 4x; **b**, 20x). The majority of tumor was comprised of bland spindle cells embedded in myxoid stroma (**c**, 4x; **d**, 20x). There were areas comprised of epitheloid tumor cells forming cord-like architecture (**e**, 10x) and with prominent atypia (**f**, 40x). There was also focal collagen rosette (**g**, 4x), and epitheloid cell whorls (**h**-**j**) with prominent atypia and rare nuclear inclusion (**j**). **h**, 10x; **i** & **j**, 40x). Focal osseous metaplasia (**k**, post decalcification, 4x) and focal perivascular condensation of bland spindle cells (**l**, 10x) were also present

Microscopic examination confirmed multinodular and infiltrative pattern (Fig. 3A, B). The majority of the tumor was consisted of regular, bland-appearing spindle cells in a fibrous and myxoid stroma (Fig. 3C, D). Thin walled elongated vessels were noted with areas of hyalinization. In certain areas the tumor cells assumed epithelioid morphology and formed a more cord-like and even whorls with more prominent cytological atypia, which is unusual for LGMFS (Fig. 3E-3J). Perivascular hypercellular areas and osseous metaplasia were present (Fig. 3K, L). Yet, no significant atypia, necrosis nor increased mitotic activity was observed.

**Fig. 4:**
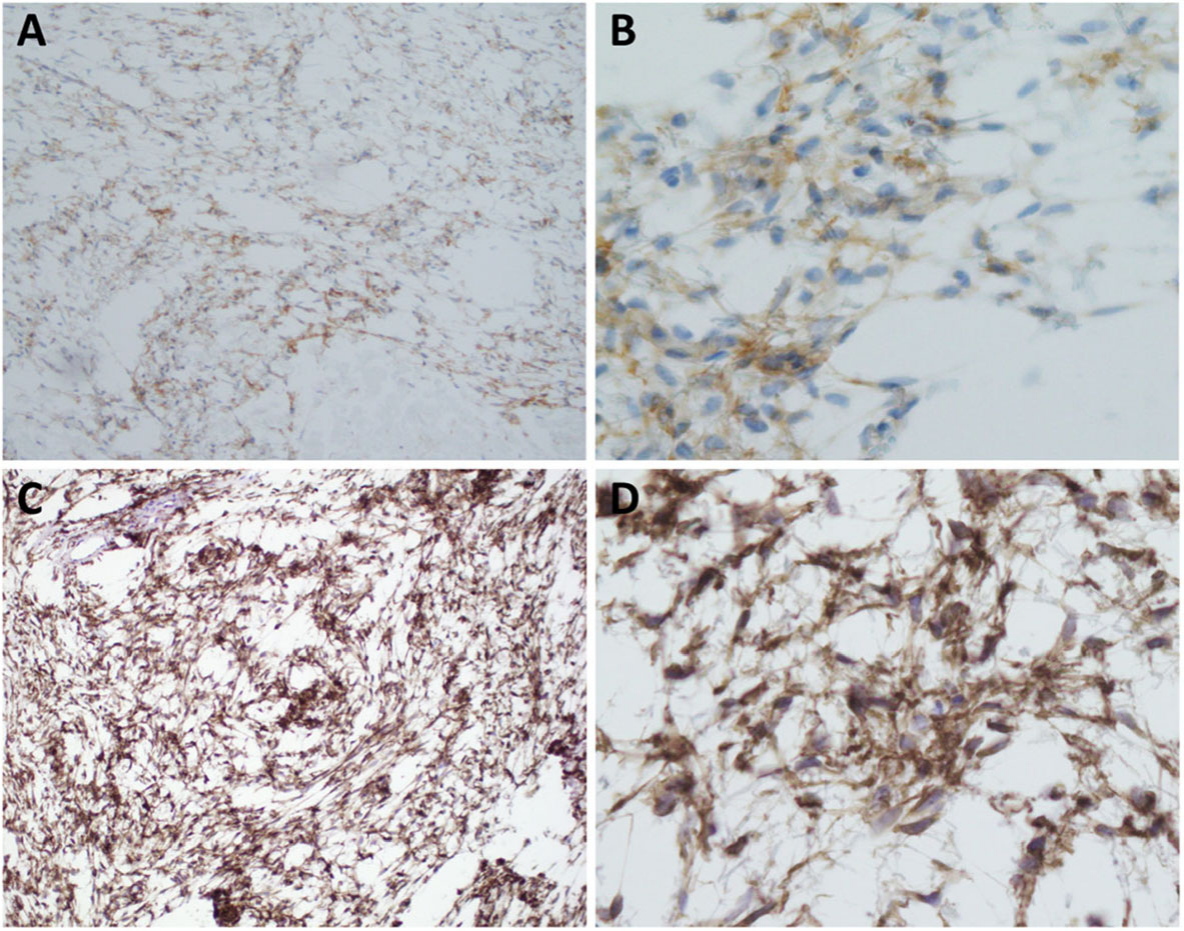
The neoplastic cells were diffusely positive for DOG1 (**a**, 10x; **b**, 40x) with moderate dense immunoreactivity, and diffusely and strongly for MUC4 (**c**, 10x; **d**, 40x)

Immunohistochemically, the tumor cells lacked of reactivity with CD34; CK5/6, EMA, SOX10, S100, desmin, SMA, MDM2 and GFAP. CD117 stain highlighted rare tumor cells. DOG1 stain showed moderate dense immunoreactivity, while MUC4 immunostain demonstrated diffuse and strong positivity in the tumor cell cytoplasm (Fig. 4).

### Molecular findings

**Fig. 5:**
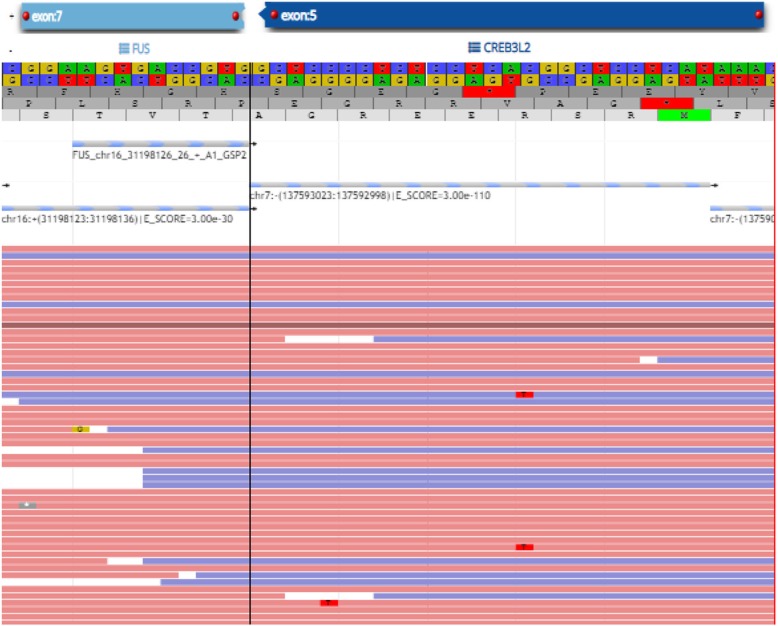
Screenshot of the Multiplexed PCR using the Archer customize panel. The image showed the fusion between FUS exon 7 and CREB3L2 exon 5. The red and blue lines referred to the different reads supporting the fusion event

Rearrangement involving FUS (16p11.2) gene was identified with break-apart probe, while rearrangement of the MDM2 and DDIT3 gene regions was not present, excluding dedifferentiated and myxoid liposarcoma. Additional confirmatory RNA from formalin fixed paraffin embedded tissue was extracted, and targeted RNA sequencing using a customized FusionPlex 86 genes panel (Archer, Boulder, CO) [28] was performed to reveal a confirmatory FUS-CREB3L2 fusion (Fig. 5).

A final diagnosis of low-grade fibromyxoid sarcoma was made based on the morphologic, immunohistochemical and cytogenetic features of the tumor.

In summary, we identified a rare case of low-grade myxofibrous sarcoma arising in small intestine mesentery. The tumor cells focally formed unusual epithelioid cords/whorls which contained prominent atypical neoplastic cells.

## Discussion

Since LGFMS is a rare tumor that can occur in a wide range of anatomical sites, it should be included in the differential diagnoses of any spindle cell tumor with low cell density. Due to its nature of hypocellularity, fine needle aspiration (FNA) biopsy can yield suboptimal amount of tissue, which may not be adequate for a complete workup to arrive at a definite diagnosis. For the present case, the deep, retroperitoneal location created extra risk of visceral organ injury and additional difficulty in obtaining enough material for further molecular and cytogenetic profiling.

The major differential diagnoses of LGFMS are mesenchymal tumors with fibromyxoid features, such as myxoma, low-grade myxofibrosarcoma, desmoid fibromatosis, nodular fasciitis, perineurioma, neurofibroma, schwannoma, ossifying fibromyxoid tumor, and dermatofibrosarcoma protuberans [13, 29]. These tumors have similar morphologic characteristics include spindle tumor cells and fibromyxoid stroma. The present case had two additional, rare but distinct, morphologic features: (1) focal metaplastic bone, and (2) foci of whorls and cord-like structures of atypical epitheloid cells. The present of bony tissue in the tumor raises the possibility of ossifying fibromyxoid tumor (OFMT), as some OFMTs can be at least focally positive for MUC4 expression. Immunostain with S100, EAAT4, INI1, MUC4 and FISH or PCR for FUS-CREB3L2 or EWSR1-CREB3L1 fusion can make the distinction, as OFMT is immunohistochemically positive for S100 AND EAATA, but negative for INI1, MUC4. It does not harbor t (7;16)(q34;p11) or t (11;16)(p11;p11) either [30].

The epithelioid foci in the present case can easily cause confusion with extraskeletal myxoid chondrosarcoma (EMCS), especially when bony tissue is present. EMCS is a biphasic neoplasm with cartilaginous foci interspersed with spindle mesenchymal cells. The tumor cells are immunostain positive for INSM1, SOX9, CD99 and S100, but negative for MUC4. They carry rearrangement of the NR4A3 but lack translocations of t (7;16)(q34;p11) or t (11;16)(p11;p11) [31, 32]. SEF, a variant of LGFMS with worse prognosis should also be taken into consideration for differential. SEF usually has large areas of hyalinized fibrous stroma and stains positive for EMA and S100 [14, 24, 33]. Synovial sarcoma (SS) can have epitheloid tumor cells as well. The tumor cells in SS are more uniform and retain t(x;18) translocation [34].

Another pitfall in the present case is gastrointestinal stromal tumours (GISTs) for three reasons. First, both of these two entities can occur in abdomen. Second, they display similar, and sometimes identical, histologic features or morphologic spectrum. Lastly, they can both show expression of DOG1 (discovered on GIST1). These similarities in location, morphology and immunophenotype can cause considerable diagnostic confusion among pathologists. Although DOG1 is a sensitive and specific marker for GIST, its expression is reported in up to 94.7% of LGFMS. More specifically, nearly 40% cases of abdomen or retroperitoneum LGFMS have variable staining positivity of DOG1 [35–37]. It should be emphasized that the differential diagnosis between these two entities relies largely on appropriate molecular profiling: LGFMS expresses MUC4 protein and harbors characteristic gene fusions. In contrast, GISTs show expression of CD117 and CD34 and are associated with Kit or PDGFRA mutations [38]. A combination of cytogenetic analysis of gene fusion and immunohistochemical staining of MUC4, CD117 and CD34 will aid in correct diagnosis.

To the best of our knowledge, our present case is the first of its kind given foci of epitheloid core and whorls and diffusely expressing DOG1. Our findings highlight the need for additional immunohistochemical and molecular studies when faced with such tumors. They also underline the importance of including LGFMS in the differential diagnosis of myxoid spindle and epithelioid DOG1+ tumors in the GI tract.

## Data Availability

The data and materials are available upon request.

## Abbreviations

DOG1: Discovered on GIST-1
EMCS: Extraskeletal myxoid chondrosarcoma
FNA: Fine needle aspiration
GIST: Gastrointestinal stromal tumours
LGFMS: Low-grade fibromyxoid sarcoma
OFMT: Ossifying fibromyxoid tumor
SEF: Sclerosing epithelioid fibrosarcoma
